# The effect of two-round presidential elections on human rights

**DOI:** 10.1371/journal.pone.0243094

**Published:** 2020-12-14

**Authors:** Joshua Holzer

**Affiliations:** Westminster College, Fulton, MO, United States of America; Sogang University (South Korea), KOREA, REPUBLIC OF

## Abstract

Recent research suggests that democratic presidential elections held using a runoff rule produce presidents that are more likely to protect human rights, in comparison to those elected under plurality rule; with this follow-up article, I seek to highlight the importance of advancing to a runoff round for those elections held using a runoff rule. I find that for presidential democracies that already have a runoff rule in place, country-years where the president has been elected after a runoff round are more likely to be associated with high government respect for human rights, in comparison to country-years where the president has been elected after only one round (that could have advanced to a runoff round, but did not). This article provides decision-makers with more information regarding the human rights consequences of runoff rounds, so that the costs and benefits of adopting (or retaining) variations of a runoff rule can be better weighed.

## Introduction

For quite some time, it has been known that different electoral rules can produce different outcomes [1–12]. More recent research has begun to explore how different electoral rules can produce different *human rights* outcomes [13]. Building upon this literature, in a recent article, I argue that a runoff rule produces presidents more likely to be associated with high government respect for human rights, in comparison to those elected under plurality rule [14]. In the end, I suggest that “this is good news for policy-makers as…simply adopting a runoff rule has the potential to improve human rights practices, even if subsequent elections do not advance to a runoff round” ([14]: 9). With this follow-up article, I seek to highlight the *importance* of advancing to a runoff round, and towards this end, I have re-estimated my previous model focusing entirely on states that utilize some form of a runoff rule. In the end, I find country-years where the president has been elected after a runoff round to be *more* likely to be associated with *high* government respect for human rights, in comparison to country-years where the president has been elected after only one round (that could have advanced to a runoff round, but did not); this suggests that policy-makers seeking to improve human rights practices through the adoption of a runoff rule should ensure that runoff rounds are not easily avoided.

## Theoretical argument

### The majority runoff rule

Since 1965, France has utilized the majority runoff rule to elect the president. Under such as rule, a *minimum* of a *majority* is required to win in the first round. If no one candidate receives at least a majority in that first round, then the top two finishers advance to a runoff election, where one of two candidates is *guaranteed* to obtain a majority. Today, majority runoff rules are common “in African countries under former French control,” in Latin American countries “in the most recent democratic period (starting with Ecuador in 1978), and in post-Communist regimes in Central and Eastern Europe since the 1990s” ([15]: 38).

As O’Neill explains, the majority runoff rule “serve[s] the seemingly noble purpose of electing a candidate with majority support, and many governments have enacted runoff voting in response to candidates winning with less than a majority of the vote” ([16]: 353). Shugart and Taagepera ([17]: 324) add that “[t]he appeal of majority runoff stems largely from a desire to avoid…the ‘Allende syndrome’: the election of a rather radical president by a narrow plurality of the vote, leading to a military dictatorship.” In Chile’s case, Allende only won roughly 36% of the vote. His unpopular presidency was then cut short when General Pinochet launched a successful coup d’état. To avoid another Allende (and the coup that followed), today Chile elects its president using the majority runoff rule. Interestingly, as part of a guided transition back to democracy, in some cases it is the “incumbent military rulers” that have introduced “majority runoff in the fear that a large but minority extreme party could take over power in an open election” ([18]: 92).

One of the downsides of the majority runoff rule is that “[h]olding a second election is expensive, the campaign season is longer, and voters must take the time to go to the polls a second time” ([16]: 352). In order to afford the longer campaign season, candidates have been know to resort to extreme measures. For instance, after failing to win a majority in the first round of the 1994 Colombian presidential election, “Ernesto Samper was accused …of having accepted money from the Cali drug cartel to finance his” runoff campaign ([19]: 158). Although he was ultimately acquitted, the scandal succeeded in damaging the legitimacy of his administration.

### The plurality runoff rule

According to Pérez-Liñán ([20]: 144), for most majority runoff elections, “the president could have been elected by plurality rule without altering the final outcome.” As a result, some states have simultaneously sought to avoid the perils of plurality rule, while also avoiding the costs associated with the majority runoff rule. This has led some states to utilized the “plurality runoff” rule to elect the president ([21]: 103). Under such a rule, a *qualifying plurality* (such as 40%) is required to win in the first round. If no one candidate receives at least that qualifying plurality in the first round, then the top two finishers advance to a runoff election. To date, plurality runoff systems are only used in Latin America, although their popularity is beginning to spread, as some scholars argue that using a “40 per cent threshold has been an unalloyed success” ([22]: 133). Currently, Argentina, Bolivia, Costa Rica, and Ecuador all use a 40% threshold in some form. Nicaragua *used* to use a variation of a 40% threshold, but has since reverted back to plurality rule. Next I’ll review Argentina’s adoption of this system, as it is illustrative of the reform process many states underwent when adopting the plurality runoff rule.

Argentina’s 1853 constitution was “[l]ike many other Latin American constitutions during the nineteenth century…inspired by the precedent of the American constitution,” and as such, utilized an electoral college to indirectly election the president ([23]: 110). In 1994, Argentina amended their constitution to eliminate their electoral college and introduce a plurality runoff system. As Kamińksi ([24]: 84) notes, during times of reform, “the selection of voting procedures” is often the result of “a bargaining process between two” conflicting parties. In Argentina’s case, the plurality runoff rule was “thus created as a compromise between the plurality rule preferred by the ascending but not dominant PJ [*Partido Justicialista* or Justicialist Party] and the majority formula preferred by the declining UCR [*Unión Cívica Radical* or Radical Civic Union]” ([25]: 430). According to Negretto ([23]: 114), “[i]nitially, the UCR demanded a threshold of 50 per cent, while the PJ stuck to a floor of no more than 40 per cent…negotiators from both parties agreed to ‘split the difference’” and came up with a novel solution: a candidate needs a qualifying plurality of at least 45% to avoid a runoff, or they can *also* avoid a runoff by netting 40% of the vote and having at least a 10-point lead over the second-place candidate. Many scholars have praised Argentina’s plurality runoff rule; Tanaka ([26]: 123), for instance, has suggested that his native country, Peru, should consider adopting a similar rule for electing the president. However, one aspect of runoff rules that few scholars have considered is the relationship between runoff rounds and government respect for human rights [27].

### Runoff rounds and human rights

Do democratic elections increase or decrease the likelihood that governments respect human rights? The answer to this question may seem intuitive, but the literature has thus far been mixed. On one side of the debate, scholars argue that elections empower a population vis-à-vis its government, which decreases the likelihood that a government will resort to repressing its own people [28–33]. This body of literature argues that by creating an “opportunity for citizen involvement in the political process,” elections “provide a motive for elected representatives to be [held] accountable to their constituents” ([34]: 205). Through elections, citizens “confront the controllers and supporters of sets of social arrangements that determine patterns of access to resources, services, status and power” ([35]: 6). Mill ([36]: 404) argues that “human beings are only secure from evil at the hands of others, in proportion as they have the power of being, and are, self-*protecting*.” One of the best ways to ensure that citizens are, indeed, ‘self-protecting’ is to given them in say in who presides over the nation by allowing them to “express a choice among the alternatives” ([37]: 71). As Steiner ([38]: 102) explains, “[b]y periodically subjecting elected officials to the approval of the electorate, [elections] help to arrest governmental violations of widely valued rights.” Thus, “[e]lections thereby serve a vital protective function” by “establish[ing] boundaries for governmental action” ([38]: 101–102).

On the other side of this debate, however, some scholars argue that elections increase political polarization by challenging the established order and threatening the political system, which can lead to an increase in human rights violations [39–44]. From this point of view, governing elites sometimes strategically violate human rights in an attempt to maintain stability and control [45–57]. This body of scholarship notes that “[m]any of the world’s longest-standing consolidated democracies, including France, the United Kingdom and the United States, have experienced periods of election violence” ([58]: 27). Indeed, while democracies are generally more inclined to protect human rights than non-democracies, even in democracies repression still occurs [59–68]. Hafner-Burton, Hyde, and Jablonski ([58]: 2) note that for many democracies, “[g]overnment-sponsored election violence—events in which incumbent leaders and ruling party agents employ or threaten violence against the political opposition or potential voters before, during or after elections—is common”.

Why are some democratic elections seemingly associated with repression, while others are not? I argue some of this variation can be explained by closely examining and comparing different *types* of elections. For instance, Richards ([69]: 648) initially found “that the presence of national elections, either executive or legislative, has no effect on government respect for human rights.” Interestingly, a few years later Richards and his co-author, Gelleny, re-examined the impact of national elections on government respect for human rights; this time, however, instead of lumping all national elections together (as had previously been common within the literature [70]), Richards and Gelleny disaggregated national elections as being either presidential elections (occurring within presidential systems) or lower-house legislative elections (occurring within parliamentary systems). In this reexamination, “lower-house national legislative elections were found to be associated with greater government respect for human rights, while presidential elections were associated with less respect for human rights” ([71]: 505).

By disaggregating ‘national elections’ as being either presidential elections in presidential systems or lower-house legislative elections in parliamentary systems, Richards and Gelleny [71] noticed that some elections appear to promote government respect for human rights, while others do not, which is something that Richards [69] was unable to see when he initially lumped all national elections together; such efforts at disaggregation could be taken further. For instance, Richards and Gelleny [71] lumped all presidential elections together, as have some subsequent scholars [58]. However, as I have alluded to above, not all presidential elections are the same; some occur over only one round, while those that utilize some variation of a runoff rule can potentially advance to a runoff round. As such, in a recent paper, I reexamined the impact of democratic presidential elections on government respect for human rights, but instead of lumping all presidential elections together (as Richards and Gelleny [71] did), I disaggregated all presidents as being elected under either plurality rule or a runoff rule. In the end, I found that “[i]n comparison to when the president is elected using plurality rule, when the president is elected using a runoff rule, that state is more likely to be associated with high government respect for human rights” ([14]: 3). Once again, however, I believe that such disaggregation could be taken further.

As is evident by my discussion above comparing the majority runoff rule versus the plurality runoff rule, not all runoff rules are the same. When comparing majority runoff and plurality runoff rules, Negretto ([23]: 116) notes that one of the main differences can “be found in the different incentives they provide for coalition-making among parties.” According to Riker ([72]: 33), candidates want to “create coalitions just as large as they believe will ensure winning and no larger.” This is because the more power a candidate negotiates away in order to *get* elected, the less power they will retain *after* the election. While the majority runoff rule “induce[s] different opposition parties to coalesce after the first round” in order to win in the runoff round, in plurality runoff systems, “opposing parties may have an incentive to coalesce before the election…to reach the minimum share” and therefore *avoid* a runoff round ([23]: 116). In other words, presidents elected after two rounds likely have had to broaden their coalitions more widely than those able to *avoid* a runoff round. I argue that this difference has human rights repercussions, as prior research has shown that “when presidents are able (or forced) to cobble together broad-based coalitions to win…their administrations are less likely (and less able) to violate human rights” ([73]: 1). To provide an illustrate on how this can play out, Freudenreich ([74]: 80) explains that in presidential systems, “the partisan composition of cabinets is largely predetermined by the bargaining and the competition before and during presidential elections.” Pérez-Liñán ([20]: 132) adds that being forced to advance to a runoff round encourages “the formation of inclusive electoral alliances before the second round,” which often forms “the basis for coalition governments.” Parallel to this, recent research has found “cabinets comprised of a higher percentage of individuals from parties other than that of the president to be associated with greater government respect for human rights” ([73]: 1). This is because when “ministers of an opposing party…find themselves in cabinet-level positions,” they “effectively wield a veto because if their president proposes a new policy (or the continuation of an old policy) that they disagree with, they can make the implementation of said policy much more onerous” ([73]: 2).

In sum, presidents elected after only one round likely have had to negotiate away less power, and thus have more power to potentially violate human rights, while presidents elected after two rounds likely had to broadly their winning coalition, oftentimes by negotiating away powerful cabinet-level positions, thus making it harder for such administrations to repress without consequence. As such, with this paper I seek to extend my previous finding that a runoff rule promotes government respect for human rights by focusing entirely on those countries that utilize a runoff rule, and disaggregating among those presidents elected after two rounds versus those elected after only one round. This leads me to my hypothesis:

I argue that country-years where the president has been elected after two rounds are *more* likely to be associated with *high* government respect for human rights, in comparison to country-years where the president has been elected after only one round (that could have advanced to a runoff round, but did not).

## Methods

### Sample

As my just stated hypothesis suggestions, this article seeks to ascertain whether presidents elected after a runoff round versus those elected after only one round (that could have advanced to a runoff round, but did not) are more likely to be associated with high government respect for human rights. Ultimately, the purpose of this article is to provide a follow-up to a previous article[14] where I argue that a runoff rule produces presidents more likely to be associated with high government respect for human rights, in comparison to those elected under plurality rule. In order to build upon my previous empirical findings, my sample for this follow-up article is modeled off that which was used in my previous study—i.e. presidential democracies with ‘democracy’ defined using Cheibub, Gandhi, and Vreeland’s Democracy versus Dictatorship (DD) dataset [75]. Although some human rights studies have opted to use either the Freedom House or Polity measures to identify which regimes are democratic, this is controversial as both Freedom House and Polity base their classification—in part—on how regimes respect human rights; therefore, using either measure would partially control for my outcome variable [76]. Poe and Tate ([59]: 856), argue that democracy “must be defined in terms that allow independent operationalization of the concept,” and in light of this advice, I (and many others [14, 27, 73, 76–84]) have opted to use Cheibub, Gandhi, and Vreeland’s [75] typology, as their DD dataset defines democracy in a way that does not incorporate state human rights practices. Per the DD dataset, a regime is considered to be a ‘democracy’ when the president is elected, the legislature is elected, there is more than one party competing in elections, and an alternation under identical electoral rules has taken place ([75]: 69).

### Dependent variables

For my primary dependent variable, I follow my previous article [14] in utilizing the Cingranelli-Richards (CIRI) Physical Integrity Rights Index [85], which is an additive nine-point index of four ordinal indicators of government respect for physical integrity rights: the rights of all human beings to be protected from torture, extrajudical killing, disappearance, and political imprisonment. *CIRI* scores ranges from ‘0’ (no respect for any of the four physical integrity rights) to ‘8’ (full respect for all of them). Whereas my previous article [14] only examined data up through 2011, thanks to the CIRIGHTS Data Project [86], *CIRI* scores are now available up through 2017.

Unlike my previous article [14], for this follow-up article I also utilize an alternative dependent variable: the Political Terror Scale (PTS), which—like *CIRI*—is coded using data from the US Department of State’s Country Reports on Human Rights Practices and Amnesty International’s Annual Report [87]. While *CIRI* scores are determined by *individually* evaluating instances of torture, extrajudical killing, disappearance, and political imprisonment (then adding together all four constituent scores), *PTS* scores are determined by *collectively* evaluating the range of the population effected by instances of torture, extrajudical killing, disappearance, and political imprisonment. States are designated a level ranging from ‘1’ to ‘5’: ‘1’ indicates that the state is under a secure rule of law, people are not imprisoned for their views, torture is rare or exceptional, and political murders are extremely rare; ‘2’ indicates that there is a limited amount of imprisonment for nonviolent political activity, torture is exceptional, and political murder is rare; ‘3’ indicates that there is extensive political imprisonment, and political murders are common; ‘4’ indicates that disappearances, torture, and political murders are all common, though state terror only affects those who interest themselves in politics; finally, ‘5’, which indicates that state-sanctioned repression has been extended to the whole population, and state leaders place no limits on the means or thoroughness with which they pursue personal or ideological goals [88].

While *CIRI* scores seeks to reflect “actual government practices” ([89]: 406), *PTS* scores aims to reflect “the ‘range’ of violence committed” ([88]: 368). Despite these differences, however, “PTS and CIRI essentially measure the same thing” ([90]: 88). As such, scholars that employ one of these indices as their dependent variable often report analogous estimations using the alternate index as a robustness check [27, 91–93]. In order to aid in comparability to *CIRI*, I have followed the literature’s trend by inverting *PTS* scores, such that higher scores now indicate greater government respect for physical integrity rights. Note that all summary statistics are presented in Table 1.

**Table 1:** Summary statistics.

Country-years where the president was elected after one round							
	Obs	Countries^◇^	Min	Mean	Mode (Freq)	Max	Std Dev
CIRI^1^	351	34	0	5.251	5 (91)	8	1.694
PTS^2^	351	34	1	3.638	4 (143)	5	0.896
Presidential power^3^	351	34	0.03	0.368	—	0.657	0.156
Civil conflict^4^	351	34	0	0.077	0 (329)	2	0.316
(Logged) population size	351	34	13.735	16.018	—	19.394	1.157
(Logged) GDP per capita	351	34	5.800	8.317	—	10.784	1.167
Country-years where the president was elected after two rounds							
	Obs	Countries^†^	Min	Mean	Mode (Freq)	Max	Std Dev
CIRI^1^	516	36	0	5.872	7 (147)	8	1.673
PTS^2^	516	36	1	3.909	4 (206)	5	0.936
Presidential power^3^	516	36	0.03	0.319	—	0.65	0.171
Civil conflict^4^	516	36	0	0.083	0 (478)	2	0.310
(Logged) population size	516	36	13.55	16.108	—	19.29	1.249
(Logged) GDP per capita	516	36	5.770	8.624	—	10.809	1.339
All country-years							
	Obs	Countries^⊕^	Min	Mean	Mode (Freq)	Max	Std Dev
CIRI^1^	867	42	0	5.621	7 (236)	8	1.708
PTS^2^	867	42	1	3.799	4 (349)	5	0.929
Presidential power^3^	867	42	0.03	0.339	—	0.657	0.167
Civil conflict^4^	867	42	0	0.081	0 (807)	2	0.312
(Logged) population size	867	42	13.55	16.071	—	19.394	1.213
(Logged) GDP per capita	867	42	5.771	8.500	—	10.809	1.280

^◇^ Argentina, Armenia, Benin, Bolivia, Brazil, Chile, Colombia, Costa Rica, Croatia, Dominican Republic, Ecuador, El Salvador, Georgia, Ghana, Indonesia, Kyrgyz Republic, Lithuania, Madagascar, Mali, Mongolia, Nicaragua, North Macedonia, Peru, Poland, Portugal, Romania, Senegal, Serbia, Sierra Leone, Timor-Leste, Ukraine, and Uruguay.

^†^Armenia, Austria, Benin, Brazil, Bulgaria, Chile, Colombia, Costa Rica, Croatia, Cyprus, Dominican Republic, Ecuador, El Salvador, Finland, France, Ghana, Guatemala, Guinea-Bissau, Indonesia, Lithuania, Madagascar, Mali, Niger, North Macedonia, Peru, Poland, Portugal, Romania, Senegal, Serbia, Sierra Leone, Slovak Republic, Slovenia, Timor-Leste, Ukraine, and Uruguay.

^⊕^The total does not add up to 42 because 28 countries are in both categories (i.e. for some years the president was elected after one round, while in other years, the president was elected after two rounds).

^1^Higher values indicate greater government respect for human rights.

^2^Values have been inverted such that higher values now also indicate greater government respect for human rights.

^3^Higher values indicate greater presidential power.

^4^0 indicates < 25 battle-related deaths, 1 indicates between 25 and 999 battle-related deaths, and 2 indicates > 1000 battle-related deaths.

### Independent variables

In order to test my hypothesis, I have constructed the following independent variable: *president elected after two rounds (vs. one round)*. To construct this variable, I consulted version 3.0 of Bormann and Golder’s Democratic Electoral Systems Around the World dataset [94, 95]. For each country-year, I looked up the most recent democratic election which brought the current president to power. If that election advanced to a runoff round, my *president elected after two rounds (vs. one round)* variable was coded as ‘1’. Otherwise, if the election that brought the president to power concluded after only one round, my variable was coded a ‘0’. As was the case for my previous article [14], all country-years where the president was not directly elected were omitted from my analysis. Note that for country-years that are election years, I have coded that year based on which president (i.e. either the outgoing or incoming) presided over the majority of that year.

Beyond my *president elected after two rounds (vs. one round)* variable, I include control variables based on those that were used in my previous article [14]; these variables take into account executive constraints, the level of civil conflict, population size, gross domestic product (GDP) per capita, and finally the previous year’s level of government repression. Since Poe and Tate’s defining study [59] (and as a result of later extensions of that study [64, 68]), it has become common practice within the human rights literature to include the types of control variables that I just mentioned. Indeed, within the literature it has now become common to refer to a model that includes these types of control variables as simply an “‘off-the-shelf’ Poe and Tate model” ([96]: 663); with this article, I seek to add to this legacy. At this point, I will discuss some of the specifics of my particular ‘Poe and Tate model’.

To begin, while many Poe and Tate models have included Polity IV’s XCONST variable [97] as its ‘executive constraints’ control [58, 71, 98], this “measure was designed to estimate executive constraints across *all* countries,” and as such, “it does not fully capture the variation within specifically *presidential democracies*” ([99]: 8). As such, I follow my previous article [14], as well as other recent articles [81, 99], in using Doyle and Elgie’s ([100]: 734) quantification of “the constitutional power of presidents,” which I refer to as *presidential power*. Values range from ‘0’ to ‘1’ with higher values indicating more powerful presidents.

Next, Poe and Tate ([59]: 859) note that “regimes are more coercive when they are involved in civil conflict.” As such, I follow my previous article [14] in including a measure of *civil conflict*, which is drawn from version 19.1 of the UCDP/PRIO Armed Conflict Dataset [101, 102]. This variable is coded as ‘0’ for each country-year with less than 25 battle-related deaths, ‘1’ for each country-year where there were between 25 and 999 battle-related deaths, and finally ‘2’ for each country-year where there were more than 999 battle-related deaths.

Continuing on, Poe and Tate models typically include measures for population size and GDP per capita, the former of which seems to be *negatively* associated with high government respect for human rights, and the latter of which seems to be *positively* associate with high government respect for human rights. My population size and GDP per capita measures both come from the World Bank [103], and as is common practice, both have been logged to correct their distributional nature.

Finally, Poe and Tate models often include a lagged dependent variable, as government repression in one year seems to influence government repression in subsequent years. However, “[b]ecause the CIRI [and PTS] variables are non-linear, a simple lagged dependent variable is less appropriate because it does not efficiently model the autoregressive trend in the data” ([104]: 12). As such, I follow Hafner-Burton [105]: 615) (and others [27, 68, 73, 99, 106]) in including a series of binary variables representing each level of the corresponding lagged dependent variable “to account for dependence across the categories of the dependent variable over time”.

### Results

In Table 2, you can see two ordered probit models that estimate *CIRI* scores and *PTS* scores in presidential democracies. Following the human rights literature [14, 27, 73, 81, 84, 99, 107–109], both of these models have robust standard errors clustered by country to address heteroscedasticity and the pooled nature of the data. As detailed above, these two regressions are modeled off the regressions I used in an earlier article [14], so as to build upon previous empirical findings; however, note that instead of examining *all* presidential democracies, to test my hypothesis, I have limited my sample to democracies that utilize either a majority or plurality runoff rule. Also note that unlike my previous article [14], for the analysis in this article, I utilize *PTS* scores as a check for robustness. Finally, note that whereas my previous article [14] only examined data from 1990 to 2011, this article examines data from 1990 to 2017. The additional 6 years are a result of recently released updates to both human rights datasets.

**Table 2:** Ordered probit estimates of CIRI scores and PTS scores in presidential democracies, 1990-2017.

Dependent variable(s)	CIRI	PTS
Independent variables		
President elected after two rounds (vs. one round)	0.265***	0.217**
	(0.077)	(0.085)
Presidential power	-0.879**	-0.990**
	(0.385)	(0.421)
Civil conflict	-0.894***	-0.813***
	(0.197)	(0.232)
(Logged) population size	-0.186***	-0.204***
	(0.053)	(0.060)
(Logged) GDP per capita	0.117**	0.171***
	(0.054)	(0.054)
Dependent variable at 0 in the previous year	0.737*	
	(0.394)	
Dependent variable at 1 in the previous year	1.829***	
	(0.584)	
Dependent variable at 2 in the previous year	1.751***	1.644**
	(0.621)	(0.715)
Dependent variable at 3 in the previous year	2.522***	3.343***
	(0.588)	(0.746)
Dependent variable at 4 in the previous year	3.264***	4.593***
	(0.601)	(0.746)
Dependent variable at 5 in the previous year	3.752***	6.244***
	(0.613)	(0.743)
Dependent variable at 6 in the previous year	4.533***	
	(0.645)	
Dependent variable at 7 in the previous year	5.320***	
	(0.678)	
Number of observations	867	867
Number of countries	42	42
Wald *χ*^2^	460.430	505.130
Prob > *χ*^2^	0.000	0.000
Log pseudolikelihood	-1130.216	-619.573

* p < 0.10,

** p < 0.05,

*** p < 0.01.

Numbers in parentheses are robust standard errors clustered by country. For CIRI, higher values indicate greater government respect for human rights. For PTS, the values have been inverted such that higher values now also indicate greater government respect for human rights.

Recall that my hypothesis is that country-years where the president had been elected after two rounds are *more* likely to be associated with *high* government respect for human rights, in comparison to country-years where the president had been elected after only one round (that could have advanced to a runoff round, but did not). As you can see in Table 2, my *president elected after two rounds (vs. one round)* variable is found to be positively associated with both high *CIRI* scores and high *PTS* scores; these relationships are statistically significant at least at the 95% level. Consistent with previous human rights scholarship, across both models all control variables are statistically significant and have signs pointing in the expected direction. For instance, *presidential power*, *civil conflict*, and *(logged) population size* are all found to be negatively associated with both high *CIRI* scores and high *PTS* scores, while *(logged) GDP per capita* and all of the binary variables for each level of the corresponding lagged dependent variable are found to be positively associated with both high *CIRI* scores and high *PTS* scores.

While the results reported in Table 2 are promising, the *substantive* effects of these models can be better illustrated through an analysis of predicted probabilities. As such, in Table 3, I report predicted probabilities (and 95% confidence intervals) for *CIRI* scores and *PTS* scores when the president is elected after two rounds versus one round. Note that these probabilities were estimated using the Clarify software package [110] and are all based upon each control variables’ mean (or mode in the case of categorical variables) for all country-years in my dataset; these values can be seen in the bottom third of Table 1.

**Table 3:** The percent change in predicted probabilities of CIRI scores and PTS scores when the president is elected after two rounds vs. one round.

	CIRI score				
	4	5	6	7	8
Elected after one round	0.018	0.135	0.300	0.454	0.093
	[0.008, 0.033]	[0.087, 0.194]	[0.253, 0.346]	[0.365, 0.541]	[0.051, 0.149]
Elected after two rounds	0.009	0.090	0.253	0.503	0.145
	[0.004, 0.018]	[0.058, 0.130]	[0.205, 0.302]	[0.416, 0.584]	[0.080, 0.229]
Difference	-0.008	-0.045	-0.047	0.050	0.052
	[-0.017, -0.003]	[-0.079, -0.017]	[-0.075, -0.021]	[0.020, 0.084]	[0.019, 0.095]
Percentage change	-48.3%	-33.6%	-15.8%	11.0%	56.2%
	PTS score				
	1	2	3	4	5
Elected after one round	0.000	0.001	0.196	0.712	0.091
	[0.000, 0.000]	[0.000, 0.004]	[0.137, 0.265]	[0.656, 0.761]	[0.054, 0.138]
Elected after two rounds	0.000	0.001	0.142	0.725	0.132
	[0.000, 0.000]	[0.000, 0.002]	[0.097, 0.197]	[0.674, 0.771]	[0.079, 0.198]
Difference	0.000	-0.001	-0.054	0.014	0.041
	[-0.000, -0.000]	[-0.002, -0.000]	[-0.099, -0.012]	[-0.008, 0.042]	[0.009, 0.080]
Percentage change	-69.0%	-50.1%	-27.4%	not significant	45.0%

95% confidence intervals are in brackets. For CIRI, higher values indicate greater government respect for human rights. For PTS, the values have been inverted such that higher values now also indicate greater government respect for human rights.

Starting with *CIRI* scores—which are reported in the top-half of Table 3—you can see that the probability of a *CIRI* score of ‘8’ (i.e. the highest possible score for government respect for human rights) is 0.093 when the president is elected after *one* round. In comparison, note that the probability of a *CIRI* score of ‘8’ is 0.145 when the president is elected after *two* rounds. Note that the difference in the probability going from 0.093 to 0.145 is 0.052, which—as you can see—is statistically significant at least at the the 95% level, given that the corresponding 95% confidence interval (which is in brackets) does not overlap with zero. Substantively, increasing a 0.093 probability by 0.052 is roughly a 56% increase. This means that for a given year, the ‘average’ state in my dataset (i.e. a state whose parameters match the mean/mode of my control variables, which can be see in the bottom third of Table 1) is roughly 56% *more* likely to be at the highest level of government respect for human rights when the president has been elected after two rounds versus one round.

Continuing on, you can see that a *CIRI* score of 7 corresponds with a positive percent change (i.e. roughly 11%), while lower scores *CIRI* scores (such as ‘4’ through ‘6’) all correspond with *negative* percent changes. Together, this suggest that a state is *more* likely to be at *higher* levels of government respect for human rights and *less* likely to be at *lower* levels of government respect for human rights when the president has been elected after two rounds versus one round. In sum, consistent with my hypothesis, presidents elected after two rounds appear to be better at protecting human rights than presidents elected after one round (that could have advanced to a runoff round, but did not). Moving to the bottom half of Table 3, you seen that this trend is robust to an alternate indicator of human rights, i.e. *PTS* scores.

## Conclusion

In a recent article, I found that a runoff rule produces presidents more likely to be associated with high government respect for human rights, in comparison to those elected under plurality rule [14] As such, I suggested that democracies with a runoff “provision would be wise to retain it, while those that have not yet adopted a runoff rule should consider doing so” ([14]: 1); with this follow-up article, I would like to amend this suggestion. I now recommend that presidential democracies adopt (or retain) runoff provisions that make advancing to a runoff round hard to avoid; this is because there appears to be human rights benefits to advancing to a runoff round, as I have found that country-years where the president has been elected after a runoff round are *more* likely to be associated with *high* government respect for human rights, in comparison to country-years where the president has been elected after only one round (that could have advanced to a runoff round, but did not).

As noted by others, “[i]t is probably not a controversial normative point to require that a good electoral method be one that does not make it likely that the winner will have been endorsed narrowly by…less than a majority of the electorate” ([17]: 328). Empirically, this article finds those administrations elected after being able to *avoid* advancing to a runoff round to be associated with worse human rights practices in comparison to those that were elected *after* a runoff round; thus, an area for future research could be to more closely examine countries that make it particularly difficult to avoid advancing to a runoff round. For instance, in Indonesia, a presidential candidate must receive more than 50% of the vote nationally *and* have at least 20% of the vote in more than half of all provinces in order to avoid advancing to a runoff round. In Sierra Leone, a presidential candidate must receive more that 55% of the vote in order to avoid advancing to a runoff round. However, as previously mentioned, increasing the likelihood of a runoff round does come with added administrative and security costs, which can be problematic for less-developed nations. As such, governments have to come to their own “conclusions as how to best trade off the costs and benefits of runoff elections” ([16]: 354). A goal of this article is to provide decision-makers with more information regarding the human rights consequences of runoff rounds, so that the costs and benefits of adopting (or retaining) variations of a runoff rule can be better weighed.

## References

[1] Rae D. The political consequences of election laws. Yale University Press; 1971.

[2] Fishburn PC. A comparative analysis of group decision methods. Behavioral Science. 1971; 16(6): 538–544. 10.1002/bs.3830160604

[3] Fishburn PC, Gehrlein WV. An analysis of simple two-stage voting systems. Behavioral Science. 1976; 21(1): 1–12. 10.1002/bs.3830210102

[4] Fishburn PC, Gehrlein WV. An analysis of voting procedures with nonranked voting. Behavioral Science. 1977; 22(3): 178–185. 10.1002/bs.3830220304

[5] Straffin PD. Topics in the Theory of Voting. Birkhauser; 1980.

[6] Nurmi H. Voting procedures: A summary analysis. British Journal of Political Science. 1983; 13(2): 181–208. 10.1017/S0007123400003215

[7] Paine NR. An Extension of Nurmi’s Summary Analysis of Voting Procedures. British Journal of Political Science. 1988; 18(2): 281–286. 10.1017/S000712340000510X

[8] Lijphart A. The political consequences of electoral laws, 1945–85. American Political Science Review. 1990; 84(2): 481–496. 10.2307/1963530

[9] Sartori G. Comparative Constitutional Engineering: An Inquiry into Structures, Incentives, and Outcomes. Palgrave Macmillan; 1994.

[10] Cox GW. Making Votes Count: Strategic Coordination in the World’s Electoral Systems. Cambridge University Press; 1997.

[11] Norris P. Electoral Engineering: Voting Rules and Political Behavior. Cambridge University Press; 2004.

[12] Ahmed A. Democracy and the Politics of Electoral System Choice: Engineering Electoral Dominance. Cambridge University Press; 2013.

[13] Cingranelli D, Filippov M. Electoral rules and incentives to protect human rights. The Journal of Politics. 2010; 72(1): 243–57. 10.1017/S0022381609990594

[14] Holzer J. Reevaluating the presidential runoff rule: Does a provision promote the protection of human rights? PLOS ONE. 2019; 14(5): e0217650 10.1371/journal.pone.0217650 31150462 PMC6544259

[15] Colomer JM. The strategy and history of electoral system choice In: Colomer JM (Ed) Handbook of Electoral System Choice. Palgrave Macmillan; 2004.

[16] O’Neill JC. Choosing a runoff election threshold. Public Choice. 2007; 131(3–4): 351–64.

[17] Shugart MS, Taagepera R. Plurality versus majority election of presidents: A proposal for a “double complement rule”. Comparative Political Studies. 1994; 27(3): 323–48. 10.1177/0010414094027003001

[18] Colomer JM. The Americas: General overview In: Colomer JM (Ed) Handbook of Electoral System Choice. Palgrave Macmillan; 2004.

[19] Dugas JC. Drugs, lies, and audiotape: The Samper crisis in Colombia. Latin American Research Review. 2001; 36(2): 157–174.

[20] Pérez-Liñán A. Evaluating presidential runoff elections. Electoral Studies. 2006; 25(1): 129–46. 10.1016/j.electstud.2005.04.002

[21] Fishburn PC, Brams SJ. Approval voting, Condorcet’s principle, and runoff elections. Public Choice. 1981; 36(1): 89–114. 10.1007/BF00163773

[22] Lehoucq F. Costa Rica: Modifying majoritarianism with 40 per cent threshold In: Colomer JM (Ed) Handbook of Electoral System Choice. Palgrave Macmillan; 2004.

[23] Negretto GL. Argentina: Compromising on a qualified plurality system In: Colomer JM (Ed) Handbook of Electoral System Choice. Palgrave Macmillan; 2004.

[24] Kamińksi MM. How communism could have been saved: Formal analysis of electoral bargaining in Poland in 1989. Public Choice. 1999; 98(1–2): 83–109.

[25] Negretto GL. Choosing how to choose presidents: Parties, military rulers, and presidential elections in Latin America. The Journal of Politics. 2006; 68(2): 421–433. 10.1111/j.1468-2508.2006.00417.x

[26] Tanaka M. Democracia sin partidos Perú, 2000–2005: Los problemas de representación y las propuestas de reforma política. Instituto de Estudios Peruanos; 2005.

[27] Holzer J. Democratic presidential elections and human rights: does a runoff round reduce repression? The International Journal of Human Rights. 2018; 22(8): 1087–110. 10.1080/13642987.2018.1499622

[28] Lipset SM. Political Man: The Social Base of Politics. Doubleday; 1960.

[29] Milnor AJ. Elections and Political Stability: An Analytic Study. Little, Brown, and Company; 1969.

[30] Rose R. Electoral Participation: A Comparative Analysis (Vol. 2). Sage; 1980.

[31] Booth JA, Seligson MA (Eds). Elections and Democracy in Central America. The University of North Carolina Press; 1989.

[32] Lewis-Beck MS. Economics and Elections: The Major Western Democracies. The University of Michigan Press; 1990.

[33] Sisk T. Democratization in South Africa: The Elusive Social Contract. Princeton University Press; 1995.

[34] Dalton RJ. Citizen Politics in Western Democracies. Chatham House; 1988.

[35] Stiefel M, Wolfe M. Citizen Politics in Western Democracies. Zed Books; 1994.

[36] Mill JS. Considerations on representative government In: Robson JH (Ed) Essays on Politics and Society. University of Toronto Press; 1977.

[37] Dahl R. Democracy and Its Critics. Yale University Press; 1989.

[38] Steiner HJ. Political participation as a human right In: Harvard Human Rights Yearbook (Volume One). Harvard Law School; 1988.

[39] Herman ES, Brodhead F. Demonstration Elections: U.S.-Staged Elections in the Dominican Republic, Vietnam, and El Salvador. South End Press; 1984.

[40] Austin D. Democracy and Violence in India and Sri Lanka. Council on Foreign Relations Press; 1995.

[41] Seligson MA, Booth JA (Eds). Elections and Democracy in Central America, Revisited. The University of North Carolina Press; 1995.

[42] Collier P. Wars, Guns, and Votes: Democracy in Dangerous Places. Harper Perennial; 2009.

[43] Robinson JA, Torvik R. The real swing voter’s curse. American Economic Review. 2009; 99(2): 310–315. 10.1257/aer.99.2.310

[44] Collier P, Vicente PC. Violence, bribery, and fraud: The political economy of elections in Sub-Saharan Africa. Public Choice. 2012; 153(1–2): 117–147. 10.1007/s11127-011-9777-z

[45] Walter E. Terror and Resistance: A Study of Political Violence. Oxford University Press; 1969.

[46] Hibbs DA. Mass Political Violence: A Cross-National Causal Analysis. Wiley; 1973.

[47] Pirages D. Managing Political Conflict. Praeger; 1976.

[48] Piven FF, Cloward RA. Poor People’s Movements: Why They Succeed, How they Fail. Vintage Books; 1979.

[49] Gurr TR. The political origins of state violence and terror: A theoretical analysis In: Stohl M, Lopez GA (Eds) Government Violence and Repression: An Agenda for Research. Greenwood Press; 1986.

[50] Ziegenhagen EA. The Regulation of Political Conflict. Praeger; 1986.

[51] Lichbach MI. Deterrence or escalation: The puzzle of aggregate studies of repression and dissent. Journal of Conflict Resolution. 1987; 31(2): 266–297. 10.1177/0022002787031002003

[52] Duvall R and Stohl M. Governance by terror In: Stohl M (Ed) The Politics of Terrorism, 3rd edition Marcel Dekker; 1988.

[53] Franks CES (Ed). Dissent and the State. Oxford University Press; 1989.

[54] Churchill W, Wall JV. The COINTELPRO Papers: Documents from the FBI’s Secret Wars Against Dissent in the United States. Oxford University Press; 1990.

[55] Karklins R, Petersen R. Decision calculus of protesters and regimes: Eastern Europe 1989. Journal of Politics. 1993; 55(3): 588–614. 10.2307/2131990

[56] Tarrow SG. Power in Movement: Social Movements, Collective Action and Politics. Cambridge University Press; 1994.

[57] Francisco RA. Coercion and protest: an empirical test in two democratic states. American Journal of Political Science. 1996; 40(4): 1179–1204. 10.2307/2111747

[58] Hafner-Burton EM, Hyde SD, Jablonski RS. When do governments resort to election violence? British Journal of Political Science. 2014; 44(01): 149–179. 10.1017/S0007123412000671

[59] Poe SC, Tate CN. Repression of human rights to personal integrity in the 1980s: A global analysis. American Political Science Review. 1994; 88(4): 853–872. 10.2307/2082712

[60] Davenport C. Multi-dimensional threat perception and state repression: An inquiry into why states apply negative sanctions. American Journal of Political Science. 1995; 39(3): 683–713. 10.2307/2111650

[61] Fein H. More murder in the middle: Life integrity violations and democracy in the world, 1987. Human Rights Quarterly. 1995; 17(1): 170–191.

[62] Krain M. State-sponsored mass murder: The onset and severity of genocides and politicides. Journal of Conflict Resolution. 1997; 41(3): 331–360. 10.1177/0022002797041003001

[63] Davenport C. Human rights and the democratic proposition. Journal of Conflict Resolution. 1999; 43(1): 92–116. 10.1177/0022002799043001006

[64] Poe SC, Tate CN, Keith LC. Repression of human rights to personal integrity revisited. International Studies Quarterly. 1999; 43(2): 291–313. 10.1111/0020-8833.00121

[65] Zanger S. A global analysis of the effect of political regime changes on life integrity violations, 1977-1993. Journal of Peace Research. 2000; 37(2): 213–233. 10.1177/0022343300037002006

[66] Harff B. No lessons learned from the Holocaust? Assessing risks of genocide and political mass murder since 1955. American Political Science Review. 2003; 37(2): 97(1): 57–73. 10.1017/S0003055403000522

[67] Davenport C, Armstrong DA. Democracy and the violations of human rights: A statistical analysis from 1976 to 1996. American Journal of Political Science. 2004; 48(3): 538–554. 10.1111/j.0092-5853.2004.00086.x

[68] Richards DL, Webb A, Clay KC. Respect for physical-integrity rights in the twenty-first century: Evaluating Poe and Tate’s model 20 years later. Journal of Human Rights. 2015; 14(3): 291–311. 10.1080/14754835.2015.1061423

[69] Richards DL. Perilous proxy: Human rights and the presence of national elections. Social Science Quarterly. 1999; 80(4): 648–665.

[70] Davenport C. From ballots to bullets: An empirical assessment of how national elections influence state uses of political repression. Electoral Studies. 1997; 16(4): 517–540. 10.1016/S0261-3794(97)00032-2

[71] Richards DL, Gelleny RD. Good things to those who wait? National elections and government respect for human rights. Journal of Peace Research. 2007; 44(4): 505–23. 10.1177/0022343307078946

[72] Riker WH. Theory of Political Coalitions. Yale University Press; 1962.

[73] Holzer J. The perils of plurality rule and the major(itarian) effect of cabinet composition on human rights in presidential democracies. Research & Politics. 2018; 5(3): 2053168018794753 10.1177/2053168018794753.

[74] Freudenreich J. The formation of cabinet coalitions in presidential systems. Latin American Politics and Society. 2016 Winter; 58(4):80–102. 10.1111/laps.12003

[75] Cheibub JA, Gandhi J, Vreeland JR. Democracy and dictatorship revisited. Public Choice. 2010; 143(1–2): 67–101. 10.1007/s11127-009-9491-2

[76] Crabtree CD, Fariss CJ. Uncovering patterns among latent variables: Human rights and de facto judicial independence. Research & Politics. 2015; 2(3): 1–9.

[77] Møller J, Skaaning SE. Autocracies, democracies, and the violation of civil liberties. Democratization. 2013; 20(1): 82–106. 10.1080/13510347.2013.738863

[78] Bellinger NM. Voting and human rights in democratic societies. Human Rights Review; 2017; 18(3): 263–282. 10.1007/s12142-017-0451-9

[79] Crabtree CD, Nelson MJ. New evidence for a positive relationship between de facto judicial independence and state respect for empowerment rights. International Studies Quarterly. 2017; 61(1): 210–24. 10.1093/isq/sqw056

[80] Conrad CR, Hill DW, Moore WH. Torture and the limits of democratic institutions. Journal of Peace Research. 2018; 55(1): 3–17. 10.1177/0022343317711240

[81] Holzer J. Measuring presidential centrism and its effect on repression: Does ideology influence whether democratic governments respect human rights? Political Science. 2018; 70(3): 253–64. 10.1080/00323187.2018.1560590

[82] Jackson JL, Hall SL, Hill DW. Democracy and police violence. Research & Politics. 2018; 5(1): 1–8.

[83] Ghatak S, Gold A, Prins BC. Domestic terrorism in democratic states: Understanding and addressing minority grievances. Journal of Conflict Resolution. 2019; 63(2): 439–467. 10.1177/0022002717734285

[84] Holzer J. When justice answers to the president: reexamining the effect of cabinet partisanship on human rights in presidential democracies. The Social Science Journal. 2020; 1–10. 10.1016/j.soscij.2019.04.006

[85] Cingranelli DL, Richards DL, Clay KC. The Cingranelli-Richards (CIRI) Human Rights Dataset, Version 2014.04.14. Available at: http://www.humanrightsdata.com/p/data-documentation.html.

[86] Cingranelli DL, Filippov M, Mark S. The CIRIGHTS Dataset, Version 2018.02.18. Available at: https://www.binghamton.edu/institutes/hri/researcher-resources.html.

[87] Gibney M, Cornett L, Wood R, Haschke P, Arnon D, Pisanò A, et al. The Political Terror Scale, 1976-2018. Available at: http://www.politicalterrorscale.org.

[88] Wood RM, Gibney M. The Political Terror Scale (PTS): A re-introduction and a comparison to CIRI. Human Rights Quarterly. 2010; 32: 367–400. 10.1353/hrq.0.0152

[89] Cingranelli DL, Richards DL. The Cingranelli and Richards (CIRI) human rights data project. Human Rights Quarterly. 2010; 32: 401–424. 10.1353/hrq.0.0141

[90] Haschke P, Gibney M. Are Global Human Rights Conditions Static or Improving? In: Backer DA, Bhavnani R, Huth PK (Eds.) Peace and Conflict 2017. Routledge; 2017.

[91] Cingranelli D, Filippov M. Electoral rules and incentives to protect human rights. The Journal of Politics. 2010; 72(1): 243–57. 10.1017/S0022381609990594

[92] Cingranelli D, Fajardo-Heyward P, Filippov M. Principals, agents and human rights. British Journal of Political Science. 2014; 44(3): 605–30. 10.1017/S0007123413000070

[93] Cingranelli DL, Kalmick C. Is Religion the Enemy of Human Rights? Human Rights Quarterly. 2019; 41(3): 725–52. 10.1353/hrq.2019.0050

[94] Bormann NC, Golder M. Democratic electoral systems around the world, 1946–2011. Electoral Studies. 2013; 32(2): 360–9. 10.1016/j.electstud.2013.01.005

[95] Bormann NC, Golder M. Democratic electoral systems around the world, Version 3.0. Available at: http://mattgolder.com/elections.

[96] Hill DW, Jones ZM. An empirical evaluation of explanations for state repression. American Political Science Review. 2014; 108(3): 661–87. 10.1017/S0003055414000306

[97] Marshall MG, Gurr TR, Jaggers K. olity IV: Political regime characteristics and transitions, 1800-2013 dataset user’s manual. Center for Systemic Peace; 2014.

[98] De Mesquita BB, Downs GW, Smith A, Cherif FM. Thinking inside the box: A closer look at democracy and human rights. International Studies Quarterly. 2005; 49(3): 439–57. 10.1111/j.1468-2478.2005.00372.x

[99] Holzer J. The effect of copartisan justice ministers on human rights in presidential democracies. PLOS ONE. 2020; 15(9): e0234938 10.1371/journal.pone.0234938 32877403 PMC7467281

[100] Doyle D, Elgie R. Maximizing the reliability of cross-national measures of presidential power. British Journal of Political Science. 2016; 46(4): 731–41. 10.1017/S0007123414000465

[101] Gleditsch NP, Wallensteen P, Eriksson M, Sollenberg M, Strand H. Armed conflict 1946-2001: A new dataset. Journal of Peace Research. 2002; 39(5): 615–37. 10.1177/0022343302039005007

[102] Gleditsch NP, Wallensteen P, Eriksson M, Sollenberg M, Strand H. UCDP/PRIO Armed Conflict Dataset, Version 19.1. Available at: https://ucdp.uu.se/downloads/.

[103] World Bank World Development Indicators, 1960-2019. Available at: https://4datacatalog.worldbank.org/dataset/world-development-indicators.

[104] Drury AC, Peksen D. Women and economic statecraft: The negative impact international economic sanctions visit on women. European Journal of International Relations. 2014; 20(2): 463–90. 10.1177/1354066112448200

[105] Hafner-Burton EM. Trading human rights: How preferential trade agreements influence government repression. International Organization. 2005; 59(3): 593–629. 10.1017/S0020818305050216

[106] Drury AC, Peksen D. Women and economic statecraft: The negative impact international economic sanctions visit on women. European Journal of International Relations. 2014; 20(2): 463–90. 10.1177/1354066112448200

[107] Dreher A, Gassebner M, Siemers LH. Globalization, economic freedom, and human rights. Journal of Conflict Resolution. 2012; 56(3): 516–46. 10.1177/0022002711420962

[108] Chilton AS, Versteeg M. Do constitutional rights make a difference? American Journal of Political Science. 2016; 60(3): 575–89. 10.1111/ajps.12239

[109] Holzer J. Nationalism and human rights: A replication and extension. PLOS ONE. 2020; 14(8): e0219409 10.1371/journal.pone.0219409 PMC6707544 31442227

[110] Tomz M, Whittenberg J, King G. Clarify: Software for interpreting and presenting statistical results. Journal of Statistical Software. 2003; 8(1): 1–30. 10.18637/jss.v008.i01

